# Preoperative lymphocyte-to-monocyte ratio predicts clinical outcome in patients undergoing radical cystectomy for transitional cell carcinoma of the bladder: a retrospective analysis

**DOI:** 10.1186/1471-2490-14-76

**Published:** 2014-09-19

**Authors:** Sally Temraz, Deborah Mukherji, Zein Al Abideen Farhat, Rami Nasr, Maya Charafeddine, Mohammed Shahait, Mohammad Rachad Wehbe, Rami Abou Ghaida, Ibrahim Abu Gheida, Ali Shamseddine

**Affiliations:** 1 Departments of Hematology-Oncology, Riad El Solh, Beirut, Lebanon; 2 General Surgery, Riad El Solh, Beirut, Lebanon; 3 Urology, and Radiation Oncology, American University of Beirut Medical Center, Riad El Solh, Beirut, Lebanon; 4 Radiation Oncology, American University of Beirut Medical Center, Riad El Solh, Beirut, Lebanon

**Keywords:** Urinary bladder neoplasms, Transitional cell carcinoma, Inflammation, Lymphocytes, Monocytes

## Abstract

**Background:**

Inflammation is a critical component of tumorigenesis, and many cancers arise from sites of infection, chronic irritation, and inflammation. Inflammatory cytokines triggered by tumors alter hematologic components, including neutrophil, lymphocyte, and monocyte counts. The neutrophil-to-lymphocyte and platelet-to-lymphocyte ratios have been shown to be valuable prognostic markers in various types of cancers, including bladder cancer. Risk stratification based on clinicopathologic data is insufficient to support treatment-related choices in patients with bladder cancer. Novel prognostic markers are therefore needed. An elevated pretreatment lymphocyte-to-monocyte ratio (LMR) is reportedly associated with improved overall survival (OS) and a longer time to treatment recurrence (TTR) in some types of cancers. However, these data are lacking in patients with bladder cancer. The aim of the present study was to investigate the effect of the preoperative LMR on OS and TTR in a cohort of patients with bladder cancer.

**Methods:**

Sixty-eight patients with transitional cell carcinoma of the bladder were included in this retrospective analysis. The associations between a high and low LMR with OS and TTR were analyzed using Kaplan–Meier curves and compared by the log-rank test.

**Results:**

In our study cohort, an elevated preoperative LMR was significantly associated with an increased TTR (P = 0.001) and OS (P = 0.020). Patients with an LMR of ≤2.87 showed a median TTR of 2.0 years (95% CI, 0.27–3.73), whereas patients with an LMR of >2.87 had a median TTR of 11.1 years (95% CI, 2.31–19.88) (P = 0.001). Patients with an LMR of ≤2.81 showed a median OS of 2.7 years (95% CI, 0.63–4.70), whereas patients with an LMR of >2.81 had a median OS of 6.0 years (95% CI, 3.60–8.40) (P = 0.020). The clinical stage at diagnosis was the only clinicopathologic feature associated with the LMR, while tumor invasion depth showed borderline significance.

**Conclusions:**

The LMR is an easily measured and inexpensive prognostic marker that was significantly correlated with OS and TTR in the present retrospective analysis. However, because of the small sample size in this study, larger multicenter, prospective studies are needed.

## Background

Inflammation is a critical component of tumorigenesis, and many cancers arise from sites of infection, chronic irritation, and inflammation [1]. It has been hypothesized that the synthesis of inflammatory cytokines triggered by the tumor microenvironment alters acute-phase reactants and hematologic components, including neutrophil, monocyte, and lymphocyte counts [1,2]. Moreover, nonsteroidal anti-inflammatory medications have been suggested to reduce the risk of developing bladder cancer by 19%, implying a critical correlation between inflammation and bladder cancer [3]. The neutrophil-to-lymphocyte and platelet-to-lymphocyte ratios have been shown to be valuable prognostic markers in patients with various types of tumors [4-9], including bladder cancer [10,11]. Risk stratification based on clinicopathologic data is insufficient to support treatment-related choices in patients with bladder cancer [12]. Novel prognostic markers that aid in stratifying patients and making treatment-related decisions are therefore necessary. However, few data regarding the preoperative lymphocyte-to-monocyte ratio (LMR) as a prognostic marker in patients with cancer are currently available. A low lymphocyte count might result in a weak, insufficient immunologic reaction to a tumor [13], while an elevated monocyte count may promote tumorigenesis and angiogenesis through local immune suppression and stimulation of tumor neovasculogenesis [14]. Hence, a low LMR may accurately reflect the presence of a weak immune response and high tumor burden.

Previous studies of hematologic malignancies suggest that a survival benefit is associated with an increased LMR [15,16]. In patients with stage III colon cancer, an increased LMR might be an independent prognostic marker of both the time to treatment recurrence (TTR) and overall survival (OS) [17]. Moreover, a decreased LMR represents an independent poor prognostic factor in patients with soft tissue sarcoma [18]. Based on these findings, we investigated the effect of the preoperative LMR on OS and TTR in a cohort of patients with bladder cancer undergoing cystectomy at our institution.

## Methods

We performed a retrospective chart review of patients diagnosed with bladder cancer at our institution from 1998 to 2007. Approval was obtained from our institutional review board before initiation of the study. The inclusion criteria were transitional cell carcinoma (TCC) and the performance of cystectomy. Patients with squamous cell carcinoma, adenocarcinoma, and clear cell carcinoma and those who did not undergo cystectomy were excluded from the analysis.

Clinical, histopathologic, and demographic features were retrospectively obtained from the patients’ medical records. Staging was performed according to the American Joint Committee on Cancer, 7th edition, 2010. The follow-up data of all patients were available from routine clinic visits to their physician. The preoperative white blood cell count and baseline complete blood cell count were routinely obtained before any interventions. Before neoadjuvant chemotherapy, the LMR was calculated from this routinely performed preoperative blood cell count as the absolute lymphocyte count divided by the absolute monocyte count. Analysis of the white blood cell count was performed in the general laboratory of our hospital.

### Statistical analysis

Receiver operating characteristic curve analysis was applied for each OS and TTR event to determine the optimal cut-off levels for the LMR as a predictor of OS and TTR. OS was defined as the duration of time between the date of the operation and the date of death of any cause, and TTR was defined as the duration of time between the date of the operation and the date of tumor recurrence. Patients who were still alive or disease-free were censored at the last follow-up date. Kaplan–Meier curves were applied to assess the correlation between the LMR and the time-to-event for OS and TTR. The log-rank test was used to determine the presence of a statistical difference in the LMR with respect to survival or disease progression between the two groups. The median times to survival and progression and their corresponding standard deviations were retrieved from the Kaplan–Meier curves. The chi-squared test was performed to identify the relationships between the LMR and survival and clinical characteristics at the time of diagnosis. All statistical analyses were performed using the Statistical Package for Social Sciences, version 20.0 (SPSS Inc., Chicago, IL, USA). A two-sided P-value of <0.05 was considered statistically significant in all tests.

## Results

The median age of the entire cohort at the time of diagnosis was 65 years (range, 43–88 years). The median follow-up duration was 2 years. Table 1 shows the baseline patient and tumor characteristics.

**Table 1:** Baseline patient and tumor characteristics

**Parameter**	**N**	**%**
Gender		
Male	60	88.2
Female	8	11.8
Smoking		
No	22	32.4
Yes	46	67.6
Lymph node density		
‹ 0.2	56	82.4
≥0.2	12	17.6
Adjuvant chemotherapy		
No	51	75.0
Yes	17	25.0
Neo-adjuvant chemotherapy		
No	65	95.59
Yes	3	4.41
Muscle invasion *(4 missing)*		
Non-muscle invasive	13	20.3
Muscle invasive	51	79.7
Grade at diagnosis		
Grade I	9	13.2
Grade II	13	19.1
Grade II	46	67.6
Stage at diagnosis		
Stage I	14	20.6
Stage II	22	32.4
Stage III	20	29.4
Stage IV	12	17.6
T Stage		
T_1_	13	19.1
T_2_	30	44.1
T_3_	16	24.5
T_4_	9	13.3
N Stage		
N_0_	52	76.5
N_1_	10	14.7
N_2_	2	2.9
N_3_	4	5.9
Parameter	Mean ± SD	
Hemoglobin (g/dL)	12.3 ± 1.72	
WBC count (10^3^/ul)	7.7 ± 1.9	
Platelets (10^3^/ul)	263.1 ± 90.7	
LN density (among patients with LN involvement)	37% (Range 12–100)	
Lymphocyte	24.9 ± 7.9	
	Range (6–46)	
Monocyte	7.0 ± 2.4	
	Range(2 – 12)	
LMR	3.5 ± 1.9	
	Range (1.38 – 11.50)	

**Figure 1:**
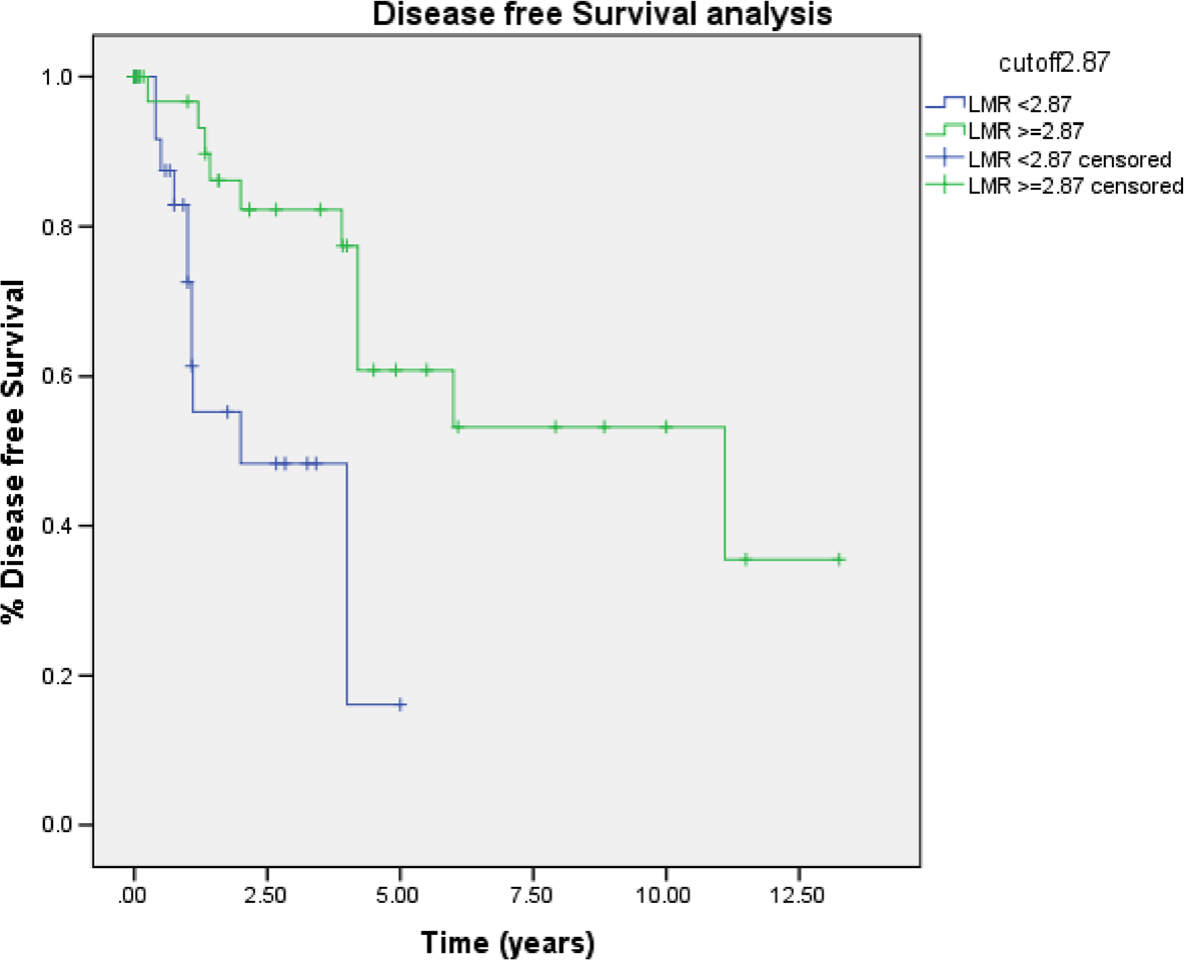
Kaplan–Meier Curves: Preoperative LMR of 2.87 and TTR in patients with TCC.

**Figure 2:**
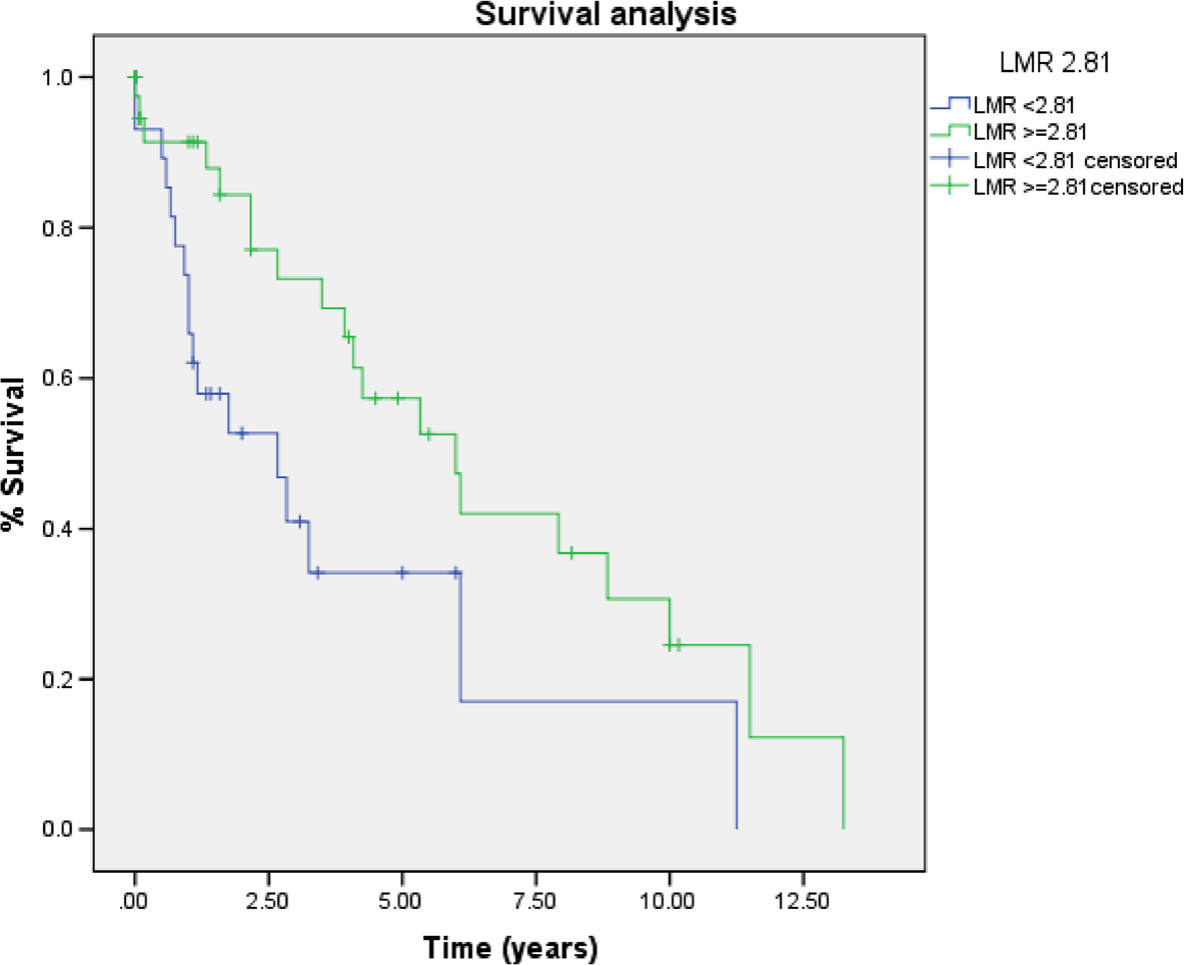
Kaplan–Meier Curves: Preoperative LMR of 2.81 and OS in patients with TCC.

Of the 68 patients with TCC bladder cancer, 23 (34%) developed disease recurrence and 37 (54%) died within the follow-up period. Receiver operating characteristic curve analysis showed that the optimal LMR cut-off level was 2.81 for OS and 2.87 for TTR. The LMR was calculated for all patients. Tumors recurred in 12 of 30 patients with an LMR of ≤2.87 and in 11 of 38 patients with an LMR of >2.87. Seventeen of 29 patients with an LMR of ≤2.81 and 20 of 39 patients with an LMR of >2.81 died. As shown in Table 2, the clinical stage at diagnosis was the only clinicopathologic feature associated with the LMR, while the tumor invasion depth showed borderline significance.An elevated preoperative LMR was significantly associated with increased TTR (P = 0.001) (Figure 1). Patients with an LMR of ≤2.87 showed a median TTR of 2.0 years (95% CI, 0.27–3.73), whereas patients with an LMR of >2.87 had a median TTR of 11.1 years (95% CI, 2.31–19.88) (P = 0.001). Patients with an LMR of ≤2.81 showed a median OS of 2.7 years (95% CI, 0.63–4.70), whereas patients with an LMR of >2.81 had a median OS of 6.0 years (95% CI, 3.60–8.40) (P = 0.020) (Figure 2).

**Table 2:** Correlation of clinical features with LMR of 2.81 (survival cut-off)

	**LMR < 2.81 (% )**	**LMR ≥2.81 (% )**	**P-value**
T Stage			
T1	5	8	0.064
T2	6	24	
T3	6	10	
T4	6	3	
Total	23 (35%)	45 (65%)	
Stage at diagnosis			
I	6	8	0.024
II	4	18	
III	11	9	
IV	8	4	
Total	29 (43%)	39 (57%)	
Grade at diagnosis			
G1	4	5	0.943
G2	5	8	
G3	20	26	
Total	29 (43%)	39 (57%)	
Lymph node density			
<0.2	22	34	0.226
≥0.2	7	5	
Total	29 (43%)	39 (57%)	

## Discussion

To the best of our knowledge, this is the first study to assess the influence of the LMR on OS and TTR in patients with TCC bladder cancer. Univariate analysis showed that an LMR of >2.87 was significantly associated with a longer TTR, while an LMR of >2.81 was correlated with better OS. The results of our study are consistent with those of other studies of various malignancies [15-18].

Bladder cancer is frequently associated with chronic or recurrent inflammation, and a high number of inflammatory cells are found at the tumor site [19]. Monocytes represent a source of multiple chemokines/cytokines that may contribute to inflammation and immune dysfunction [20]. Monocytes reportedly promote tumorigenesis and angiogenesis through local immune suppression and stimulation of tumor neovasculogenesis [14]. Moreover, macrophages, which are differentiated monocytes, reportedly support tumor-associated angiogenesis and tumor cell invasion, migration, and intravasation; they may also lead to suppression of antitumor immune reactions [21,22]. This could explain why an elevated monocyte count confers a negative prognosis in patients with solid tumors [23,24].

Lymphocytes, on the other hand, are essential in antitumor reactions of the immune system through induction of tumor cell apoptosis. They also serve as mediators of antibody-dependent, cell-mediated cytotoxicity [25,26]. The numbers of T and natural killer cells are reportedly significantly lower in patients with invasive bladder carcinoma than in patients with superficial carcinoma [27]. Moreover, in another study, the CD4/CD8 ratio, lymphocyte reactivity to mitogens, and natural killer cell activity were significantly lower in patients with invasive disease than in controls and patients with superficial carcinoma [28]. The same study showed that patients with high-grade tumors also had a significantly lower CD4/CD8 ratio and lymphocyte activity toward mitogens than did patients with low-grade tumors [28]. Hence, a low lymphocyte count might be responsible for a weak, insufficient immunologic reaction to a tumor and could incur a negative prognostic outcome [13].

In the present study, the only factor associated with the LMR was the stage at diagnosis; the tumor depth showed only borderline significance. The tumor grade did not have a significant effect on the LMR. Because an advanced stage at diagnosis has been shown to incur a negative clinical outcome in the majority of tumors and was the only feature associated with the LMR in this study, it is possible that a low LMR reflects an advanced stage at diagnosis. However, this conclusion needs further validation in other trials.

The limitations of this study include the small sample size, which hindered appropriate utilization of multivariate analysis, and thus the ability to produce definite conclusions. Moreover, patients were not evaluated for active inflammation at the time of the complete blood cell count; thus, potential confounding factors such as infection and other disease states such as ischemia, acute coronary syndrome, diabetes, and renal and hepatic dysfunction might have affected the lymphocyte and monocyte counts.

The LMR constitutes a low-cost predictive biomarker of the clinical outcome in patients with TCC bladder cancer and is related to a patient’s adaptive immune response. Univariate analysis revealed a significant correlation between the TTR and LMR; however, use of the LMR as an independent prognosticator requires further evaluation in a large-scale study involving multivariate analyses.

## Conclusions

Invasive bladder cancer remains a challenge to oncologists and a burden to patients, who exhibit low survival rates and high tumor recurrence rates. The results of this study reveal a significant association of the LMR with both OS and TTR. However, because of the small number of patients included in this study, our results need to be properly evaluated in a cohort large enough to perform valid multivariate analyses.

## Competing interests

The authors declare that they have no competing interests.

## Authors’ contributions

ST, RN, and AS conceived of the study and participated in its design and data interpretation. DM participated in the interpretation of the data and helped in drafting and editing the paper. MC and ZF carried out the statistical analysis and participated in interpretation of the data. MS, MW, RA, and IA participated in the design and coordination of the study and helped to review the paper. All authors read and approved the final manuscript.

## Pre-publication history

The pre-publication history for this paper can be accessed here:

http://www.biomedcentral.com/1471-2490/14/76/prepub
